# Using patient values and preferences to inform the importance of health outcomes in practice guideline development following the GRADE approach

**DOI:** 10.1186/s12955-017-0621-0

**Published:** 2017-05-02

**Authors:** Yuan Zhang, Pablo Alonso Coello, Jan Brożek, Wojtek Wiercioch, Itziar Etxeandia-Ikobaltzeta, Elie A. Akl, Joerg J. Meerpohl, Waleed Alhazzani, Alonso Carrasco-Labra, Rebecca L. Morgan, Reem A. Mustafa, John J. Riva, Ainsley Moore, Juan José Yepes-Nuñez, Carlos Cuello-Garcia, Zulfa AlRayees, Veena Manja, Maicon Falavigna, Ignacio Neumann, Romina Brignardello-Petersen, Nancy Santesso, Bram Rochwerg, Andrea Darzi, Maria Ximena Rojas, Yaser Adi, Claudia Bollig, Reem Waziry, Holger J. Schünemann

**Affiliations:** 1 Department of Health Research Methods, Evidence and Impact, McMaster University, 1280 Main Street West, Hamilton, ON L8S 4K1 Canada; 2 Iberoamerican Cochrane Centre, CIBERESP-IIB Sant Pau, Barcelona, Spain; 3 Department of Medicine, McMaster University, Hamilton, Canada; 4 Department of Internal Medicine, Faculty of Health Sciences, American University of Beirut, Beirut, Lebanon; 5 Cochrane Germany, Medical Center - University of Freiburg, Faculty of Medicine, University of Freiburg, Freiburg, Germany; 6 Centre de Recherche Épidémiologie et Statistique Sorbonne Paris Cité – U1153, Inserm/Université Paris Descartes, Cochrane France, Hôpital Hôtel-Dieu, 1 place du Parvis Notre Dame, 75181 Paris, Cedex 04 France; 7 Evidence-Based Dentistry Unit, Faculty of Dentistry, Universidad de Chile, Santiago, Chile; 8 Departments of Internal Medicine/Nephrology and Biomedical and Health Informatics, University of Missouri-Kansas City, Kansas City, MO USA; 9 Department of Family Medicine, McMaster University, David Braley Health Sciences Centre, 100 Main Street West, 6th Floor, Hamilton, ON L8P 1H6 Canada; 10 School of Medicine, University of Antioquia, Medellín, Colombia; 11 Tecnologico de Monterrey School of Medicine, Monterrey, Mexico; 12 Ministry of Health, Riyadh, Saudi Arabia; 13 Division of Cardiology, Department of Medicine, Veterans Affairs Medical Center, Buffalo, NY USA; 14 Department of Internal Medicine, University at Buffalo, the State University of New York, Buffalo, NY USA; 15 Hospital Moinhos de Vento, Porto Alegre, Brazil; 16 National Institute of Science and Technology for Health Technology Assessment, Federal University of Rio Grande do Sul, Porto Alegre, Brazil; 17 Department of Internal Medicine, Pontificia Universidad Católica de Chile, Santiago, Chile; 18 Department of Clinical Epidemiology and Biostatistics, Pontificia Universidad Javeriana, Bogotá, Colombia; 19 King Faisal Specialist Hospital and Research Centre, Riyadh, Saudi Arabia; 20 The Kirby Institute, University of New South Wales, New South Wales, Australia

**Keywords:** Patient values, Patient preferences, Outcome importance, Systematic review, Guideline development, Evidence to decision

## Abstract

**Background:**

There are diverse opinions and confusion about defining and including patient values and preferences (i.e. the importance people place on the health outcomes) in the guideline development processes. This article aims to provide an overview of a process for systematically incorporating values and preferences in guideline development.

**Methods:**

In 2013 and 2014, we followed the Grading of Recommendations Assessment, Development and Evaluation (GRADE) approach to adopt, adapt and develop 226 recommendations in 22 guidelines for the Ministry of Health of the Kingdom of Saudi Arabia. To collect context-specific values and preferences for each recommendation, we performed systematic reviews, asked clinical experts to provide feedback according to their clinical experience, and consulted patient representatives.

**Results:**

We found several types of studies addressing the importance of outcomes, including those reporting utilities, non-utility measures of health states based on structured questionnaires or scales, and qualitative studies. Guideline panels used the relative importance of outcomes based on values and preferences to weigh the balance of desirable and undesirable consequences of alternative intervention options. However, we found few studies addressing local values and preferences.

**Conclusions:**

Currently there are different but no firmly established processes for integrating patient values and preferences in healthcare decision-making of practice guideline development. With GRADE Evidence-to-Decision (EtD) frameworks, we provide an empirical strategy to find and incorporate values and preferences in guidelines by performing systematic reviews and eliciting information from guideline panel members and patient representatives. However, more research and practical guidance are needed on how to search for relevant studies and grey literature, assess the certainty of this evidence, and best summarize and present the findings.

**Electronic supplementary material:**

The online version of this article (doi:10.1186/s12955-017-0621-0) contains supplementary material, which is available to authorized users.

## Background

According to the World Health Organization (WHO), “a recommendation [in a practice guideline] tells the intended end-user of the guideline what he or she can or should do in specific situations to achieve the best health outcomes possible, individually or collectively…” [1]. A recommendation does not only depend on the magnitude of an intervention effect, but should incorporate other considerations and criteria that determine the direction and strength of a recommendation, such as the importance or weight of the health outcomes [2]. Recommendations are the deliberate product of inclusively considering these criteria that influence decision-making by a multidisciplinary group through a structured process [3–6]. This multidisciplinary group typically includes content experts, patients, methodologists and other stakeholders [7–9]. These different individuals may choose different treatment options when they are presented with the same evidence. When full understanding of the information is ensured, different choices for recommendations are often the result of disparate values and preferences.

Although infrequently practiced, ideally this information should be based on evidence from thoroughly conducted research, which is collected through a systematic approach [10]. The main reason for incorporating values and preferences in guideline development process is that recommendations aligned with patient values and preferences may be more easily accepted, implemented and adhered to by those intended to benefit from the guidelines. Additionally, in the individual physician-patient encounters, recommendations with consideration of patient’s preferences, can better inform the decision-making process [10–15]. Further motivations for incorporating patient values and preferences in guideline include ethical and moral imperatives, accountability and legitimacy of the guideline developers.

The Grading of Recommendation, Assessment, Development and Evaluation (GRADE) working group developed the Evidence-to-Decision (EtD) framework to facilitate the process of integrating the criteria considered necessary in guideline development and documenting such process for different audiences [4, 5]. With this framework, to formulate a recommendation, these criteria include: balance between desirable and undesirable effects, certainty in the evidence informing the recommendation, resource utilization, and impact on health system equity, feasibility of the recommendation, stakeholder acceptability, patient values and preferences. A number of tools and initiatives explicitly describe the factors that should be considered when developing recommendations with different stakeholders. These include the development of the Guidelines International Network (GIN)-McMaster Guideline Development Checklist [3], the presentation methods developed in GRADE’s Developing and Evaluating Communication Strategies to Support Informed Decisions and Practice Based on Evidence (DECIDE) Project [16] as well as collaborative guideline development activities with professional and governmental organizations. However, we still recognize paucity in practical strategies to incorporate patient values and preferences in the guideline development process.

In fact, the definition of and strategies for determining values and preferences are still under debate. The GRADE approach includes the consideration of patient values and preferences as the relative importance of outcomes or health states of interest [2, 3, 17, 18]. Similarly, in health economics, preference is a general term that includes health utilities elicited under uncertainty (e.g. results from standard gamble), as well as the values elicited under certainty (e.g. time trade off or visual analogue scale) [19–21]. With this GRADE definition, the preference for or against an intervention is conceptually equivalent to the importance placed on outcomes that follow from the decision to undergo an intervention. That is, the preference for or against an intervention is a result of indirectly weighing the health outcomes it causes (e.g. the outcome burden when taking a medication or the consequences of undergoing surgery such as the outcome postoperative pain) [3]. Thus, the preference for or against a treatment intervention is an implicit result of the relative importance of the health outcomes an individual connects to the intervention. However, while values and preferences directly relate to the relative importance of health outcomes in practice guidelines, they also implicitly relate to achieving better health outcomes when judging other aspects that are relevant for a decision. These other aspects such as attitudes, expectations and beliefs are also considered under this umbrella term [22, 23]. In the GRADE EtD, these aspects often fall within other criteria of the EtD framework (e.g., equity, feasibility or acceptability considerations). For example, if a society places low value on avoiding resource expenditure for wide implementation of a new intervention, it may be considered feasible. Patients may find an intervention administered by a health worker other than a physician not acceptable, if they expect the latter to administer it. Thus, feasibility and acceptability are considerations related to values and preferences but not as directly related to the importance patients place on the health outcomes.

Box 1. Relevant criteria in Evidence-to-Decision Framework

**Table:** 

People values and preferences: the relative importance people place on the health outcomes; since we consider an intervention in the context of the consequences it incurs, the preferences for or against an intervention is a consequence of the relative importance people place on the expected or definite health outcomes it incurs.	
Acceptability and feasibility: views or perspectives or importance of health outcomes placed by stakeholders beyond the target population of the recommendation	

Despite the increasing importance of practice guidelines in the management of health problems, there is a lack of evidence informing about initiatives using values and preferences in the guideline development process. Therefore, we addressed the challenges of integrating values and preferences in practice guidelines. Generally, we utilized the GRADE system for guideline development that is endorsed by over 100 organizations and applied worldwide [10]. Specifically, we first developed an approach for systematically identifying information on values and preferences. Second, we conducted case studies on how to consider local values and preferences evidence in the guideline development process. Our case studies were based on 22 guidelines with 226 recommendations covering diverse clinical areas in a new national guideline program for the Ministry of Health of Saudi Arabia.

## Methods

**Fig. 1:**
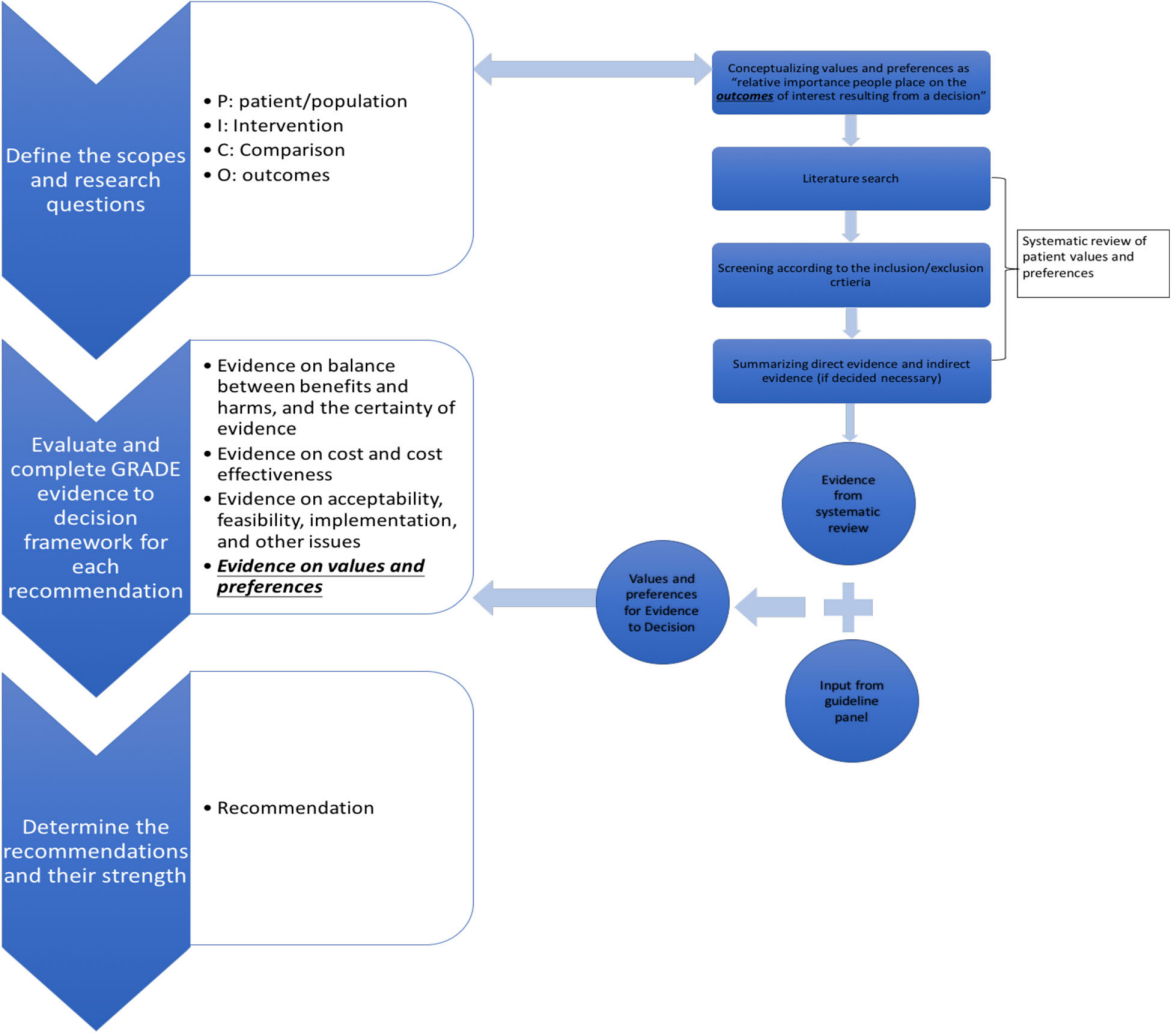
Process of Integrating Values and Preferences. The steps on the left show the process of integrating values and preferences in guideline development. The guideline panel formulated the recommendations based on evidence on values and preferences, together with other evidence, e.g., evidence on the balance between benefits and harms and cost

For these guidelines, we were specifically interested in identifying values and preferences relevant to the context of the Saudi society. Methodological details of the guideline development process for the Saudi Ministry of Health are described elsewhere [24, 25]. The Ministry of Health of Saudi Arabia had embarked on standardizing and coordinating guideline development nationally to promote the awareness and practice of evidence-based medicine [24, 25]. In this project, we used the definition of “relative importance of outcomes” for patient values and preferences. We undertook several steps to obtain information about patient values and preferences. We performed a systematic review to summarize relevant studies of values and preferences in populations of interest. In addition, we sought input from clinical experts and consulted patient representatives (see the Fig. 1). To assess the feasibility of our approach, we also monitored the workload resulting from conducting systematic reviews on values and preferences during guideline development.

### Systematic review

Our approach to comprehensively identifying and understanding existing evidence about values and preferences started with a systematic review summarizing the relevant research evidence [26]. Similar to any systematic review process this included formulation of research questions, literature search, screening according to eligibility criteria, as well as appraisal and summary of the available evidence [7, 14, 27].

#### Formulation of research question and GRADE definition of values and preferences

We defined the values and preferences as the relative importance of outcomes and formulated the research question for the systematic review of values and preferences as: “what is the relative importance that a population of interest places on the main outcomes?” With this research question, we considered both the studies on the relative importance of outcomes and studies on the preferences for or against an intervention eligible in the 22 guidelines and the detailed recommendations therein.

#### Eligibility criteria

Studies reporting the “relative importance of outcomes” relevant to the guideline disease topics were included. We included studies that elicited utilities of outcomes through direct measurement techniques including standard gamble, time trade off, visual analogue scales (VAS), and indirect measurement techniques based on generic tools such as EuroQol five dimensions questionnaire (EQ-5D), HUI (health utility index), QWB (quality of wellbeing), as well as utility or health status values transformed (mapping) from quality of life measurement [28–30]. We recognize that not all scientists consider VAS a utility instrument because it does not include a choice under uncertainty. While acknowledging this, we consider VAS measures as eligible to indicate the relative importance of outcomes. Direct choice refers to the technique of asking participants to choose from a set of options. We included studies that expressed the preferences through willingness to pay, probability trade off, discrete choice exercise, ranking, and paired comparison. We also included studies that used other questionnaires and scales, sometimes self-developed to ask preference for outcomes. We also included studies that measured the importance of outcomes in qualitative studies (See Table 1) [23, 31]. Eligible studies included either participants who were experiencing the relevant health states or participants who did not experience the health state of interest but were provided with descriptions of scenarios of the health state [32–34].

**Table 1:** Eligibility criteria for the systematic review of patient values and preferences

Category	Measurement
Utility/Health Status Value	Standard Gamble
	Time Trade Off
	Visual Analogue Scale
	Multi-attribute instruments (i.e. EQ-5D utility, HUI utility)
	Utility or health status values transformed (mapping) from quality of life measurements (both generic or disease specific tools) ^a^
Non-utility, quantitative information	Direct/Forced Choice exercise: choice from a set of options
	Non-utility measurement of health states: other self-developed questionnaires and scales
Qualitative information	Qualitative research

^a^ Referring to transforming scores from quality of life measurement into a utility or health status value based on transformation equations

#### Literature search

We conducted 22 systematic reviews on information suggesting the importance of outcomes; one for each guideline. We developed a broad search filter for values and preferences studies for Ovid Medline, EMBASE and PsychInfo, informed by a search strategy utilized in a previous guideline development process [14]. This search filter included keywords for the following concepts: *health state values, preference, utility, attitude to health, patient decision, patient participation, patient satisfaction, patient view, patient perception* and their variant formats so as to be as inclusive as possible and capture all potential relevant studies (see Additional file 1). The development of the search strategy is another ongoing project and the detailed development process will be reported in another publication [Selva A, Solà I, Zhang Y, Sanabria AJ, Pequeño S, Rigau D, Martínez L, Mas G, Haynes RB, Schünemann HJ, Alonso-Coello P: Development and use of a content search filter for studies on how patients and other stakeholders value health outcomes (submitted)].

In order to address local values and preferences and enhance contextual information, we also added a geographic search filter that restricted the search to the Kingdom of Saudi Arabia and more broadly to the Middle East. Thus, we developed a complex search strategy based on three search filters: a broad values and preferences filter, the disease specific filters for each guideline, and a geographic filter. These filters were combined using a Boolean “AND”.

#### Screening and data abstraction

We systematically screened titles and abstracts and retrieved studies for full text screening if they were deemed eligible or if the abstract lacked the detail to determine eligibility by at least one of the screeners. We reviewed the full text articles and summarized the findings stratified according to Table 1 and incorporated them into the GRADE EtD frameworks for each of the 22 guideline areas. We a priori broadened our inclusion criteria and included indirect evidence from other settings when we did not identify information specific for the Saudi Arabia setting.

### Input from panel members

Furthermore, we asked guideline panel members (including patient representatives with and without previous experience in the condition of interest) to provide their views on the relative importance of the main outcomes, and their experience related to the disease of interest. We specifically asked clinicians to reflect on patients’ views based on their previous clinical interactions with patients. However, we did not conduct *de novo* studies on eliciting values and preferences for these guidelines.

## Results

### Findings of the systematic reviews

We identified a wide variety of eligible studies using utility elicitation, non-utility estimates from questionnaires or scales, as well as qualitative research. Due to heterogeneity of designs and outcomes, we did not pool results and thus provided narrative summaries of the results for each topic. We summarized the information in EtD frameworks for each panel to consider and allow for them to provide feedback. Here, we present guideline-specific examples of the identified studies to illustrate our findings. They are based on a description by utility tools that were used in the original studies.

#### Utility based estimates

For the antithrombotic guidelines that we produced, utilities for severe, moderate and mild nonfatal intracranial bleeds were identified ranging from 0.10 to 0.51, 0.29 to 0.77 and 0.47 to 0.94, respectively [35, 36]. The utility was 0.63 for nonfatal pulmonary embolism, and 0.44 to 0.84 for major bleed. A systematic review on breast lump-related values and preferences reported the following utilities: 0.96 for disease-free survival, 0.76 to local-regional recurrence, 0.72 to contralateral breast cancer and 0.64 to distant metastasis [35].

#### Non-utility measurements

For the guideline on management of breast lump and primary breast cancer, the systematic review identified one study reporting an additional year in life expectancy or 3% in survival rates was sufficient to make adjuvant chemotherapy worthwhile for 68–84% of women [35].

#### Qualitative findings

For the guideline on the screening and treatment of precancerous lesions for cervical cancer prevention, we identified one qualitative research study suggesting that women fear screening and may have a high level of anxiety related to colposcopy or treatment [35].

#### Input from panel members

Our consultations with panel members suggested that they were not aware of any studies that were missed by our systematic review process. We also asked them to indicate if indirect evidence from other settings is applicable to the Saudi Arabia setting. Generally, the panellist did not believe there were significant differences except in a few cases. For example, for breast cancer screening, the panel members suggested that in the Saudi Arabia setting, patients place a lower value for any psychological effect of false positive results and frequency of screening compared to the perceived benefits of screening strategies on mortality. In the venous thromboembolism (VTE) treatment guideline development, panellists reflected that oral anticoagulation requires frequent testing and monitoring, diet and medication restrictions, stoppage for procedures. However, anticoagulation would be given for a relatively limited period of time and patients would view potential reduction in mortality and symptomatic VTE favourably [35].

In the allergic rhinitis guideline, panel members suggested that some patients in Saudi Arabia would not accept sublingual immunotherapy with some allergens of animal origin. The panel evaluating hemodialysis options described that: “the preference to delay dialysis may be stronger in Saudi patients compared to non-Saudi patients (i.e. Saudi patients are more hesitant/resistant to start dialysis)” [35].

### Use of the information as part of decision-making process

The importance patients place on outcomes influences the balance of benefits and harms thereby impacting on the direction and strength of a health recommendation. Thus, being explicit about the relative importance requires a transparent description of how they influenced the recommendation. The panels were made aware that, following the GRADE approach, high variability or uncertainty about the values and preferences typically leads to weak or conditional recommendations [10].

Table 2 summarizes some examples showing how the guideline panels used the information when formulating recommendations. Panels were instructed to use the information provided about the relative importance of the main outcomes and balance of the desirable and undesirable consequences. Panellists also made judgments about the variability and uncertainty about the values and preferences information.

**Table 2:** Sources of information and how it was used by panels

Source of information	What is the information?	How can it be used?
Update of prior systematic review	*Utility estimate* Nonfatal Intracranial Bleed (severe): 0.1 to 0.51 Nonfatal Intracranial Bleed (moderate): 0.29 to 0.77 Nonfatal Intracranial Bleed (mild): 0.47 to 0.94 Nonfatal Pulmonary Embolism: 0.63 Major Bleed: 0.44 to 0.84 *“This result suggested intracranial bleed overall was 2 to 3 times worse than major bleed or pulmonary embolism.”*	To help guideline panellists weigh the benefits (absolute reduction in pulmonary embolism) and harms (absolute increase in bleedings).
Systematic review	*Non-utility estimate* For the guideline on management of breast lump and primary breast cancer, the systematic review identified one study reporting an additional year in life expectancy or 3% in survival rates were sufficient to make adjuvant chemotherapy worthwhile by 68–84% of women.	To judge to what extent women are willing to accept the burden of adjuvant chemotherapy to benefit from a specific amount of increased survival
Systematic review	Qualitative finding *“Evidence from qualitative studies suggested women may fear screening and may have a high level of anxiety related to colposcopy or treatment.”*	To suggest what are the views of local women on cervical cancer screening tests in relation to its psychological impact
Panel members (either physicians or patients)	*Panellists experience* In some guideline topics, *patient inputs corroborated the panel’s perception*.	To serve as complementary sources in addition to the information from systematic review.

For example, for the antithrombotic guideline, the systematic review on utilities suggested that major bleeding was equivalent to nonfatal pulmonary embolism; while intracranial bleed overall was 2 to 3 times worse than major bleed or pulmonary embolism [36]. In the Breast Lump guidelines we found that recurrence and metastasis are the most important outcomes for women, and were considered as such by the panel [35].

### How consideration of local values and preferences influenced recommendations

The presumption that local values and preferences differ from those obtained in other settings, questions the usefulness of using the latter. In several cases, local values and preferences contributed significantly to the formulation of recommendations. For example, the allergic rhinitis management guideline stated since “there is important variability about how much people value its ([sublingual immunotherapy, SLIT)] effectiveness because there is a concern that some patients in Saudi Arabia would not accept SLIT with some allergens of animal origin”. Consequently, the recommendation was a weak recommendation suggesting sublingual immunotherapy for treatment of adults with seasonal or intermittent allergic rhinitis based on moderate quality evidence [35]. Although the recommendation was not different from the source guideline [37], one of the main reasons for this weak recommendation was the expression of local patient values and preferences described above.

The recommendation comparing ultrasonography versus mammography, as part of the triple assessment of palpable breast masses in women aged 30–40 years, was associated with very low certainty in the evidence of effects. The panel suggested “patients would likely favour the use of ultrasonography” because mammography can be more painful and uncomfortable for patients. In the panels’ view this consideration of values and preferences justified a strong recommendation because ultrasonography showed better diagnostic accuracy (sensitivity and specificity) compared with mammography despite very low certainty in the evidence [35].

### Workload related to values and preferences

Incorporating values and preferences in guideline development required resources on the following levels: literature searches, screening and synthesis, preparation of the GRADE EtD frameworks and consideration of values and preferences in decision-making. During development of the search strategy, we noted that many relevant studies were difficult to identify because of the lack of a validated filter or of standardized keywords (Medical Subject Headings: MeSH terms) being used to tag eligible studies. With the definition, measurement and methodology of values and preferences for guidelines still under debate, our aim to not miss relevant information was time and resource consuming. We managed this burden by limiting our search strategy through the stepwise use of a geographic search filter when required. For example, in the Migraine Headache guidelines, we first applied a geographic filter. After identifying no eligible studies, we felt it was necessary to spend additional time and resources to do a larger search for indirect evidence outside of the local context.

Panels recognized the importance of explicitly incorporating the information in the process and considered it in all of the 226 recommendations. The structured summary and presentation of the values and preferences information for each question in the GRADE EtD framework facilitated the process of considering this type of evidence.

## Discussion

We describe an approach for the incorporation of the relative importance of health outcomes in healthcare recommendations. We applied a multi-faceted approach utilizing a systematic review strategy complemented by other information sources. We use illustrative examples to show the usefulness of identifying relevant studies and using their findings in drafting the recommendations.

### Strengths and limitations

The systematic and transparent approach to identify and summarize published literature on values and preferences is the strength of the proposed strategy. The feedback from experienced panel members suggested that we did not miss important relevant studies. A second strength is our pre-conceived and structured approach to incorporate both published and elicited local values and preferences in the decision making process. Guideline developers can assume an international or national, or, alternatively, a localized or specific perspective. By considering the appropriate setting the recommendations could potentially be more acceptable to stakeholders. While the former strategy would be helpful for international organizations such as WHO, those adapting recommendations to a specific setting should consider locally relevant evidence, as was the case in this project [1, 38].

This study has some weaknesses. While the study is based on the development of over 20 guidelines and over 200 recommendations, it is restricted to one geographic setting. Also, limited local information was identified for patient values and preferences. The one related advantage is identifying the necessity of conducting more research on local values and preferences. Second, our definition and eligibility criteria for values and preferences were broad. The inclusion of a variety of study designs resulted in challenges with determining the eligibility of individual studies and the category they belong to. The time and resources spent on systematic reviews of values and preferences varied across guideline topics. We also did not formally assess the certainty or the quality of the evidence in the values and preferences from published studies. As for information about values and preferences from panel members, the collected information was unsystematic, potentially biased, and sometimes difficult to use. Furthermore, we were not able to assess publication bias due to the nature of the study question, study design and the geographic filter we used. While we identified studies with a variety of designs providing relevant evidence, the lack of standardized methods for reporting and identifying the evidence places additional limitations on current guideline development but not on our work.

### How to interpret and present information about values and preferences in guidelines

Although the integration of values and preferences is considered standard for trustworthy guideline development processes, using systematic reviews to identify values and preferences in a structured approach is still uncommon [1, 7, 12, 39, 40]. The Saudi Arabian panels weighted the relative importance of outcomes using information from literature reviews, the panel members themselves, and patient representatives. This facilitated adoption, adaptation and creating new recommendations according to local values. The GRADE EtD framework helped facilitate the use of values and preferences information in the decision making process by explicitly calling attention to the criterion when balancing benefits and harms. The approach we used has face validity because the panel members did not identify missing studies on local values and preferences. As guideline methodology is refined, how to define, measure, and incorporate patient values and preferences will evolve.

There are other guideline efforts that consider patient values and preferences in the process of developing recommendations. For example, the National Institute for Health and Clinical Excellence (NICE) also considers the impact of values and preferences on the strength of recommendation. The process includes asking patient representatives to reveal their experience in addition to reviews of qualitative research evidence and cross-sectional surveys. However, NICE does not operationalize values and preferences as the importance of outcomes [39].

Thus, despite recently increasing numbers of available primary studies and systematic reviews on values and preferences [41–44], they are still rarely used in guidelines. This is likely also a result of poor guidance and definitions for how to incorporate this information appropriately. Our study provides a feasible approach to consider patient values and preferences in guideline development. However, other challenges in using this information remain. This includes accepted approaches to assessing the quality or certainty of evidence which is recognized by the GRADE working group and work is ongoing to develop an approach [31, 45–47]. Furthermore, existing systematic reviews seldom have a clear definition, valid search strategy, or transparent synthesis methods to identify evidence about the relative importance of outcomes. Our experience of using GRADE EtD frameworks, that do not yet routinely include modeling based on preferences, need to be seen in the context of other approaches that routinely include modeling [10, 48].

## Conclusions

Although considering the relative importance of health outcomes is essential in informing healthcare decision-making, use of this type of information remains a complex area to integrate. Our experience shows that guidelines in general and GRADE EtD frameworks in particular, lend themselves to the incorporation of this aspect in clinical and public health recommendations. To further facilitate this process a methodologically rigorous and consistent approach for reporting, summarizing and interpreting the information is needed due to the great heterogeneity on the definition, perspective and measurement of values and preferences. We provide an empirical approach to address this concern through systematic reviews and panel members’ input.

## Additional file

Additional file 1: Search strategy. (DOCX 15 kb)

## Abbreviations

DECIDE: Developing and Evaluating Communication Strategies to Support Informed Decisions and Practice Based on Evidence
EtD: Evidence-to-Decision
GIN: Guidelines International Network
GRADE: Grading of Recommendations Assessment, Development and Evaluation
HUI: Health utility index
MeSH: Medical Subject Headings
NICE: National Institute for Health and Clinical Excellence
QWB: Quality of wellbeing
SLIT: Sublingual immunotherapy
VAS: Visual analogue scales
VTE: Venous thromboembolism
WHO: World Health Organization
