# Supplementary material for: Using patient values and preferences to inform the importance of health outcomes in practice guideline development following the GRADE approach
Source: Health Qual Life Outcomes. 2017 May 2;15:52. doi: 10.1186/s12955-017-0621-0 (PMC5412036; doi:10.1186/s12955-017-0621-0)
Supplement: Additional file 1: — Search strategy. (DOCX 15 kb) [file 12955_2017_621_MOESM1_ESM.docx]

**Appendix**

Search strategy:

Values and preferences terms

1. patient$ participation.mp. or exp patient participation/

2. patient$ satisfaction.mp. or exp patient satisfaction/

3. attitude to health.mp. or exp Attitude to health/

4. (patient$ preference$ or patient$ perception$ or patient$ decision$ or patient$ perspective$ or user$ view$ or patient$ view$ or patient$ value$).mp.

5. (patient$ utilit$ or health utilit$).mp.

6. health related quality of life.mp. or exp "quality of life"/

7. (health stat$ utilit$ or health stat$ indicator$ or (health stat$ adj 2 valu$)).mp. or exp Health Status Indicators/

8. 1 or 2 or 3 or 4 or 5 or 6 or 7

Geographic terms:

1. Saudi Arab$.mp,in. or Saudi Arabia/

2. Riyadh.mp,in.

3. Jeddah.mp,in.

4. Kh*bar.mp,in.

5. Dammam.mp,in.

6. 1 or 2 or 3 or 4 or 5

7. Kuwait$.mp,in. or Kuwait/

8. United Arab Emirates.mp,in. or United Arab Emirates/

9. Qatar$.mp,in. or Qatar/

10. Oman$.mp,in. or Oman/

11. Yemen$.mp,in. or Yemen/

12. Bahr*in$.mp,in. or Bahrain/

13. 7 or 8 or 9 or 10 or 11 or 12

14. Middle East$.mp,in. or Middle East/

15. Jordan$.mp,in. or Jordan/

16. Libya$.mp,in. or Libya/

17. Egypt$.mp,in. or Egypt/

18. Syria$.mp,in. or Syria/

19. Iraq$/ or Iraq.mp,in.

20. Morocc$.mp,in. or Morocco/

21. Tunisia$.mp,in. or Tunisia/

22. Leban$.mp,in. or Lebanon/

23. West Bank.mp,in.

24. Iran$.mp,in. or Iran/

25. Turkey/ or (Turkey or Turkish).mp,in.

26. Algeria$.mp,in. or Algeria/

27. Arab$.mp,in. or Arabs/

28. 14 or 15 or 16 or 17 or 18 or 19 or 20 or 21 or 22 or 23 or 24 or 25 or 26

29. 27 or 28

30. 6 or 13 or 29
